# Gait Shear and Plantar Pressure Monitoring: A Non-Invasive OFS Based Solution for e-Health Architectures

**DOI:** 10.3390/s18051334

**Published:** 2018-04-25

**Authors:** Cátia Tavares, M. Fátima Domingues, Anselmo Frizera-Neto, Tiago Leite, Cátia Leitão, Nélia Alberto, Carlos Marques, Ayman Radwan, Eduardo Rocon, Paulo André, Paulo Antunes

**Affiliations:** 1 Instituto de Telecomunicações, Campus Universitário de Santiago, Aveiro 3810-193, Portugal; fatima.domingues@ua.pt (M.F.D.); catia.leitao@ua.pt (C.L.); nelia@ua.pt (N.A.); carlos.marques@ua.pt (C.M.); aradwan@av.it.pt (A.R.); pantunes@ua.pt (P.A.); 2 Department of Physics & I3N, University of Aveiro, Campus Universitário de Santiago, Aveiro 3810-193, Portugal; tmpl@ua.pt; 3 CSIC-UPM, Ctra. Campo Real, Arganda del Rey 28500, Madrid, Spain; e.rocon@csic.es; 4 Telecommunications Laboratory, Electrical Engineering Department, Federal University of Espírito Santo, Espírito Santo 29075-910, Brazil; frizera@ieee.org; 5 Department of Electrical and Computer Engineering and Instituto de Telecomunicações, Instituto Superior Técnico, University of Lisbon, Lisbon 1049-001, Portugal; paulo.andre@lx.it.pt

**Keywords:** gait analysis, e-Health application, physical rehabilitation, shear and plantar pressure sensor, biaxial optical fiber sensor, multiplexed fiber Bragg gratings

## Abstract

In an era of unprecedented progress in sensing technology and communication, health services are now able to closely monitor patients and elderly citizens without jeopardizing their daily routines through health applications on their mobile devices in what is known as e-Health. Within this field, we propose an optical fiber sensor (OFS) based system for the simultaneous monitoring of shear and plantar pressure during gait movement. These parameters are considered to be two key factors in gait analysis that can help in the early diagnosis of multiple anomalies, such as diabetic foot ulcerations or in physical rehabilitation scenarios. The proposed solution is a biaxial OFS based on two in-line fiber Bragg gratings (FBGs), which were inscribed in the same optical fiber and placed individually in two adjacent cavities, forming a small sensing cell. Such design presents a more compact and resilient solution with higher accuracy when compared to the existing electronic systems. The implementation of the proposed elements into an insole is also described, showcasing the compactness of the sensing cells, which can easily be integrated into a non-invasive mobile e-Health solution for continuous remote gait monitoring of patients and elder citizens. The reported results show that the proposed system outperforms existing solutions, in the sense that it is able to dynamically discriminate shear and plantar pressure during gait.

## 1. Introduction

Between 2015 and 2050, the world’s population aged over 60 years is expected to double from about 12% up to 22% (up to about 2 billion), with the group aged 80 years and over growing most rapidly (predictably will quadruple from approximately 100 million to 434 million people) [1]. Many elderly and patient groups experience varying degrees of mobility impairments, which require closer monitoring. Assistive devices play a pivotal role in their lives and have a great impact on their ability to live independently and perform basic daily tasks. The assistive products market is set to expand significantly in response to the ageing population and disability trends, with a global market for home medical equipment expected to grow from $27.8 billion in 2015 to nearly $44.3 billion by 2020 [2]. This growing demand for e-Health solutions will improve healthcare services and quality of life by providing autonomy and mobility during daily activities.

Non-invasive continuous monitoring of an individual’s health conditions, rehabilitation status, or assistance appears as a natural evolution of current healthcare services by providing patients with continuous remote support when required while guaranteeing autonomy and free mobility. Following this direction and towards improving the quality of life of physically impaired citizens by increasing their mobility, our team has been working on different practical solutions for the continuous remote monitoring of patients [3,4,5].

The monitoring and analysis of the shear and plantar pressure involved in gait is crucial for the evaluation of patients under physical rehabilitation processes, as well as for the control of rehabilitation exoskeletons in order to correct abnormal plantar pressures due to the uneven load distribution resulting from poor foot sensitivity [6,7]. Moreover, shear in particular, plays a major role in the diagnosis of foot ulceration in diabetic patients. The existence of shear forces presupposes friction between the skin-foot and the shoe. An abnormal increase of shear forces in a given plantar area can cause callosities or the so-called pressure ulcers. This health condition can occur when the tissue is compressed under pressure during gait/walk. Diabetic patients tend to lose sensitivity in the extremities of the body and the feet are one of the most affected areas. By losing sensitivity in the foot plantar areas, the patient involuntarily begins to modify the gait pattern and to adopt less correct postures that lead to the appearance of wounds, which due to their insensitivity in most cases are discovered late. An early discovery of irregularities in the gait pattern of individuals at risk is the first step in reducing the occurrence of ulceration and its treatment. Although shear stress has been identified as a pathogenic factor in the development of plantar ulcers, due to a lack of validated shear stress sensing devices, only studies related to plantar pressures are widely reported [6]. During the last few decades some methods have been proposed for the measurement of plantar shear stress [6], nonetheless, there is a lack of systems able to accurately monitor shear and plantar pressure simultaneously during gait. The work reported in this paper intends to fill such a gap while providing an ambulatory solution based on state-of-the-art optical fiber sensing technology able to be integrated as an enabler in e-Health architectures.

As a first step, we have developed a non-invasive solution for the continuous remote monitoring of foot plantar pressure during gait (walking). Our previous efforts have concentrated on pressure distribution through a strategically placed network of optical fiber sensors (OFSs) [3,4,5]. In the present work, we take a step forward by presenting the design and implementation of a fiber Bragg grating (FBG)-based platform for the simultaneous measurement of shear force (F_S_) and vertical force (F_V_), which can be useful in various applications in addition to e-Health. The proposed architecture comprises a compact and accurate biaxial OFS-based on two in-line FBGs (FBG1 and FBG2) placed individually in two adjacent cavities. For the demodulation of the optical signal registered by the designed optical sensing cell, a system of two equations was used, correlating the sensitivities of both FBGs with the F_V_ and F_S_ forces [8]. Moreover, we also present the design and integration of the sensing architecture in an insole for continuous monitoring of F_S_ and F_V_ (from which is calculated the plantar pressure), during the gait movement of patients.

The rest of this paper is organized as follows. Section 2 provides a survey of the state-of-the-art technology in monitoring shear and vertical forces, highlighting the advantages of the proposed solution. Section 3 introduces the design and calibration of the sensors and the implemented experimental protocols. Section 4 showcases the implantation of five sensing cells in an insole for gait analysis purposes. Section 5 discusses a potential e-Health architecture based on optical fiber sensors for gait analysis. The conclusion is drawn in Section 6.

## 2. Related Work

Vertical force sensors are nowadays required for a wide number of applications in diverse areas such as industrial production and structural health monitoring, artificial intelligence, robotic exoskeletons, and other health applications [3,4,5,9,10,11,12,13,14,15,16]. There are several types of F_V_ sensors, which are characterized essentially by the transduction mechanisms and technology used for converting forces into electrical signals, such as piezo resistivity and capacitance [11,14,15,16]. In addition to these electronic mechanisms, other transduction methods, such as OFSs are frequently used for the measurement of these type of forces [3,4,5,17].

Apart from the F_V_ sensors, devices with additional sensing properties, such as temperature and shear, are highly desired in equipment for medical applications. Specifically, sensors capable of simultaneously measuring F_V_ and F_S_ are highly required for the haptic perception of robotic hands, prosthetic skin, and to monitor the stress under the foot to prevent its ulceration [3,4,5,6,18,19,20,21,22,23].

Several studies have been published, using different technologies for the simultaneous sensing of F_V_ and F_S_, namely strain gauge technology [21,24], piezoelectric materials [22,25], capacitive sensors [26], micro strip antennas, and coils [27] to name a few. All these types of sensors have the great disadvantage of using electricity at the point of contact with the user of the equipment, non-immunity to electromagnetic radiation, and thus require the use of several electric cables, usually one for each sensor cell, when multiple points are monitored simultaneously. As an example of the drawbacks presented by electronic devices, the sensor developed by Chen et al. can only be used as a static equipment because, despite having a small detection area of 1.9 cm × 1.9 cm, the overall structure of the sensor is very large [24]. In the work presented by Heywood et al., problems with the electrical insulation of the four layers that constitute the sensor to avoid the short circuits were reported [26]. Also, the work developed by Moahmmad et al. has limitations on the maximum pressure that it can withstand, which is about 0.25 psi [27]. In this context, OFSs appear as an alternative technology to sense these variables, with several advantages over their electronic counterparts. Such advantages include their immunity to electromagnetic interference, remote operation and sensing capability, small dimensions, lightweight, and geometrical versatility, making the technology increasingly used as sensing devices in several areas, with special significance in the biomedical engineering and biomechanics areas [3,4,5,9,28,29].

Although few, there are already some works reporting the development of optical fiber based F_V_ and F_S_ sensors. In 2000, Koulaxouzidis et al. demonstrated that three optical fibers with one FBG each (one on the horizontal and the other two on the diagonals), embedded in a block of solid elastomer, could be used for the measurement of in-shoe shear stress [30]. The Bragg wavelength shift was found to be almost linear under shear stress, in the range between −120 kPa and 120 kPa, yielding to a sensitivity of 4.35 pm/kPa. In 2013, Zhang et al. used a similar method to produce a sensor for the measurement of the same parameters. However, in this case only two optical fibers with FBGs were used (one on the horizontal and the other on the diagonal directions), embedded in a soft polydimethylsiloxane matrix [8]. The sensitivities achieved were 0.82 pm/Pa for vertical pressure and 1.33 pm/Pa for shear. Moreover, in 2005, Wang et al. used a different sensing mechanism, consisting of an array of optical fibers, lying in perpendicular rows and columns separated by elastomeric pads [31]. In their design, the measurements of plantar and shear pressures are based on intensity attenuation in the fibers due to physical deformation. The pressure measurement relies on the force induced light loss from the two affected crossing fibers, while the shear measurement depends on relative position changes in these pressure points between the two fiber mesh layers. This method was later used by other researchers [32,33,34], where they tried to improve the quality of the obtained data, but still with the disadvantage of a high number of fibers for each measuring point, hence its high complexity and large sensor size.

Although some previous efforts reported the use of OFSs, all those works have used complex designs with more than one optical fiber, which increases their fragility and lowers their application feasibility.

With the aim of reducing the complexity of the sensing device without compromising its performance, we present the design and implementation of an FBG-based sensor for simultaneous measurement of F_V_ and F_S_. Our proposed solution stands out from previously reported ones because of the minimalism of the sensor structure and its accurate feedback. In an insole with several points of analysis it is important to use the least invasive technology possible, to reduce the amount of fiber inside the insole (with a limited size and thickness), and decrease the number of fracture and possible rupture points along the fiber. As this detection method has the ability to multiplex several sensors in the same fiber, it was possible to design an insole with several points of analysis using only one optical fiber.

## 3. Sensing Cell Design and Implementation

### 3.1. Sensing Cell Design

In the designed architecture, we used a sensing system comprising two multiplexed FBGs with a 2 mm-length, FBG1 and FBG2, spaced by 9 mm, inscribed in a photosensitive optical fiber (GF1, Thorlabs^®^ Newton, NJ, USA), using the phase mask method with a UV KrF pulsed excimer laser (Bragg StarTM Industrial-LN, Coherent, Dieburg, Germany) operating at 248 nm. A 5 mJ pulse energy was applied with a repetition rate of 500 Hz. The central Bragg wavelength of the FBGs is 1560.9 nm and 1557.6 nm, for FBG1 and FBG2, respectively.

**Figure 1:**
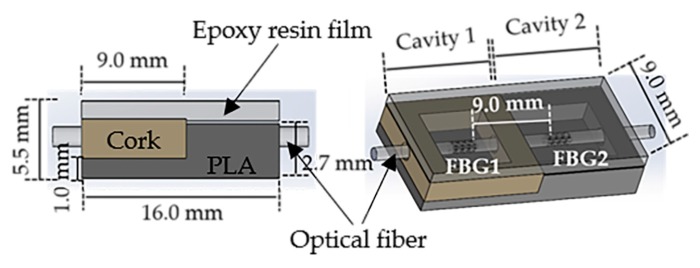
Schematic illustration of the designed sensing cell for simultaneous vertical forces (F_V_) and shear forces (F_S_) measurement.

The optical fiber containing the multiplexed FBGs was incorporated in a small cell (9.0 mm × 16.0 mm × 5.5 mm) composed by two cavities, as shown in Figure 1. The cavity in which the FBG1 was placed (cavity 1) was mechanically isolated with a cork wall with a thickness of 2 mm, while the cavity containing the FBG2 (cavity 2) was designed and 3D printed with a hard polymer (polylactic acid, PLA) with a 1.2 mm thick wall. To protect the optical fiber and provide the necessary robustness to the sensing cell, both cavities were then filled with a thermosetting epoxy resin [17], which becomes a semi-rigid structure bounded to the optical fiber, after the curing process. Despite the epoxy resin stiffness, the applied F_V_ and F_S_ still induce deformation in the cell area and consequently in the optical fiber sensors without compromising their feedback. It should be noted that no bonding points were added in the cross section between the optical fiber and the cavities’ boundaries. In that way, a vertical force applied in the cell top area will compress the epoxy resin vertically, inducing the stretching of the fiber embedded in its interior. On the other hand, a horizontal force, applied along the longitudinal axis of the fiber (left to right on the image), will compress the resin and the fiber containing the sensors against the PLA hard wall.

Due to its near zero Poisson coefficient [35], the cork walls in cavity 1 provide the necessary mechanical isolation from lateral forces (applied out of the sensing cell area), while, simultaneously, offering the necessary elasticity for the FBG1 to be actuated under vertical forces and longitudinal shear stresses.

Additionally, in order to induce different sensitivities in the FBGs, the walls of cavity 1 were designed to be slightly higher than the walls of cavity 2 (gap of 0.8 mm), and in that way, the response obtained from FBG1 can be enhanced when compared with the FBG2, since the latter is more concealed due to the hard polymer wall. To make the contact area uniform, a 2 mm thick layer of epoxy resin was placed on the top of the sensing cell, as shown in Figure 1.

### 3.2. Calibration and Performance Testing Methodology

The optical sensing cell feedback is processed in terms of the Bragg wavelength shift (∆λ). The dependence of this parameter with the strain variations (Δ*ε*) can be translated by Equation (1) [4]:

Δ*λ* = *K_ε_*.Δ*ε* (1)

where *K_ε_* is the sensor sensitivity to strain variations.

**Figure 2:**
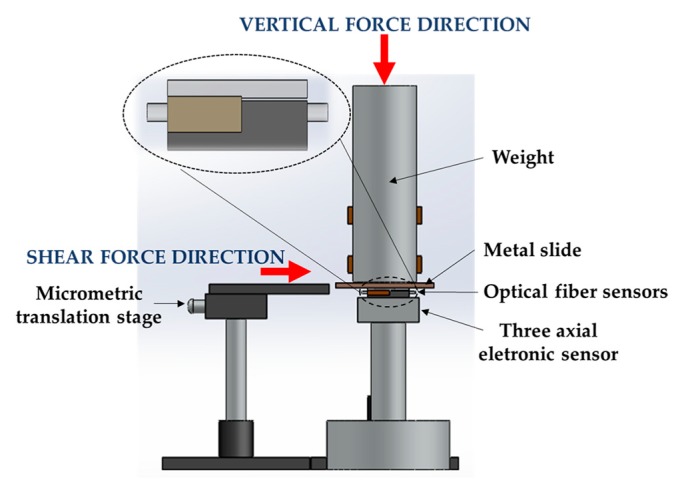
Representation of the experimental setup for the calibration and testing of the fiber Bragg grating (FBGs) cell.

For the demodulation of the reflected optical signal, it was necessary to calibrate each FBG, independently, to F_V_ and F_S_. To do so, a 3-axial electronic force sensor, composed of one biaxial (MBA400, Futek, Irvine, CA, USA) and one uniaxial (TPP-3/75, Transdutec, Barcelona, Spain) unit was used. The designed optical fiber based sensing cell (designated hereinafter as FBGs cell) was firmly attached to the three-axial sensing unit in order to guarantee that any perturbation induced in the FBGs cell would be also registered by the electronic sensing mechanism. Figure 2 is a schematic representation of the experimental setup implemented.

The data retrieved from the electronic sensor was acquired through an analog-to-digital converter (USB-6008, National Instruments, Austin, TX, USA), while the optical signal given by the FBGs cell was acquired by an interrogation system (I-MON 512 USB, Ibsen, Farum, Denmark).

For the calibration of the FBGs cell to F_S_, a metal slide placed between the sensing units and a metal cylinder bar (3 kg) was horizontally dragged with the help of a translation stage [8], as shown in Figure 2. The translation stage pushed the metal slide parallel to the sensors’ top area, inducing an F_S_ in both sensing units (electronic and optical). During this test, the F_V_ was maintained constant (∆F_V_ ≈ 0 N). For the calibration to F_V_, a variable force was applied on the cylindrical bar, while the F_S_ was kept constant (∆F_S_ ≈ 0 N). During these procedures (F_S_ and F_V_ calibration), the values registered by both sensing systems were simultaneously acquired for further comparison/calibration.

After the calibration, in order to evaluate the FBGs performance under the simultaneous application of F_V_ and F_S_, the procedures described previously were performed simultaneously: the metal slide was propelled horizontally while an F_V_ was applied in the cylindrical bar, as seen in Figure 2. During the implementation of this protocol, both sensors (electronic and optical) were simultaneously acquiring the data modulated in the sensing units. The obtained results are presented and discussed in the next subsections.

Also, the system hysteresis was tested, by inducing increasing and decreasing vertical forces in the sensing cell.

### 3.3. Calibration Results

**Figure 3:**
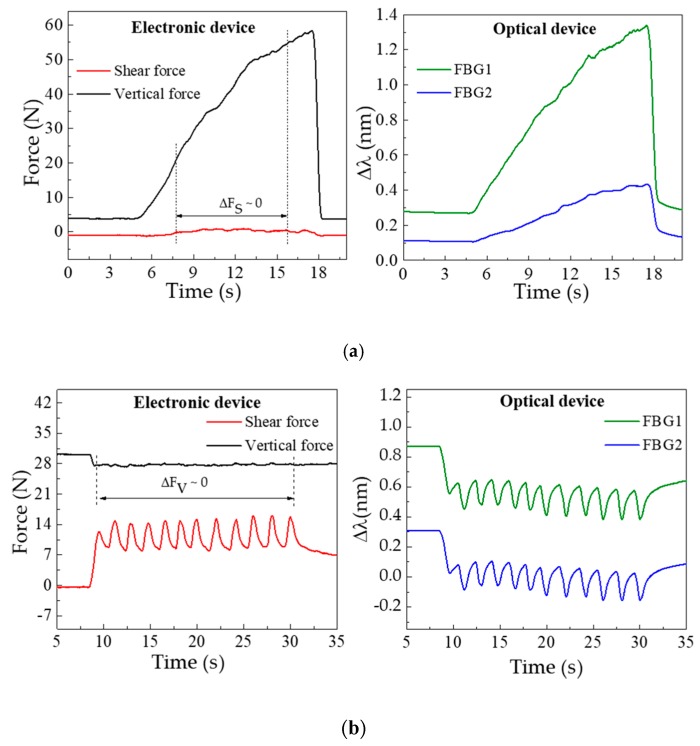
Data acquired by the three-axial electronic (left) and optical fiber (right) based systems, for the (**a**) vertical and (**b**) shear forces characterization.

Figure 3 shows the data simultaneously acquired by the electronic and optical systems, for the F_V_ (a) and F_S_ (b) characterization procedures. The F_V_ characterization, Figure 3a, clearly shows the increase of the registered pressure with the load applied over time. In the case of the optical sensor, this increase is translated by a continuous wavelength shift towards higher wavelengths in both FBGs. Such a shift is caused by the longitudinal distension (stretching) of the resin under vertical compression, which will induce the elongation of the embedded optical fiber and consequently the positive Bragg wavelength shift. In the representation of the shear calibration data (see Figure 3b), the periodic variations induced by the translation stage movements are visible in both sensors.

In the electronic device, the increasing force corresponds to the movement of the translation stage given by one complete turn of the micrometric screw (360°). Once that turn is complete there is a relaxing moment till the new turn is started, which corresponds to the decrease (return to initial state) of the applied force. In the represented characterization process, there is a total of 12 turns. In the optical sensor response, this data is inverted, hence the shear applied in the cell will longitudinally compress the resin and the embedded optical fiber, resulting in a negative Bragg wavelength shift.

**Figure 4:**
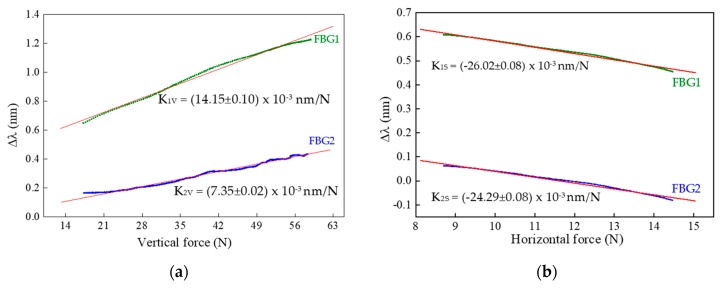
Calibration data obtained for FBG1 and FBG2 during the variation of the applied forces: (**a**) vertical (with ∆F_S_ ≈ 0 N) and (**b**) shear (with ∆F_V_ ≈ 0 N). Symbols are the acquired data and the red line corresponds to the linear fit (R^2^ > 0.99).

From the characterization procedures, the sensitivities of FBG1 and FBG2 were calculated for both the F_V_ and F_S_ applied. Towards that, for each value registered by the three-axial electronic sensor, the correspondent Bragg wavelength shift (given by the optical sensor) was correlated, as presented in Figure 4.

From the calibration representation, a linear dependence of the Bragg wavelength shift with the applied force is verified. The sensitivities obtained for FBG1 and FBG2 as a function of the vertical (K_1V_ and K_2V_) and shear forces (K_1S_ and K_2S_) were:

K_1V_ = (14.15 ± 0.10) × 10^−3^ nm/N, K_1S_ = −26.02 ± 0.08) × 10^−3^ nm/N,
K_2V_ = (7.35 ± 0.02) × 10^−3^ nm/N, K_2S_ = (−24.29 ± 0.08) × 10^−3^ nm/N.

The substantial discrepancy in the vertical force sensitivity values obtained for the two FBGs is due to the height difference between the walls of cavity 1 and 2, since there is a gap of 0.8 mm between the wall of cavity 2 and the top of the cell. However, once the fiber is not fixed in any point of the cell, its longitudinal movements are similarly transmitted to FBG1 and FBG2, and therefore their sensitivities to shear forces are not as different as that of the vertical forces.

**Figure 5:**
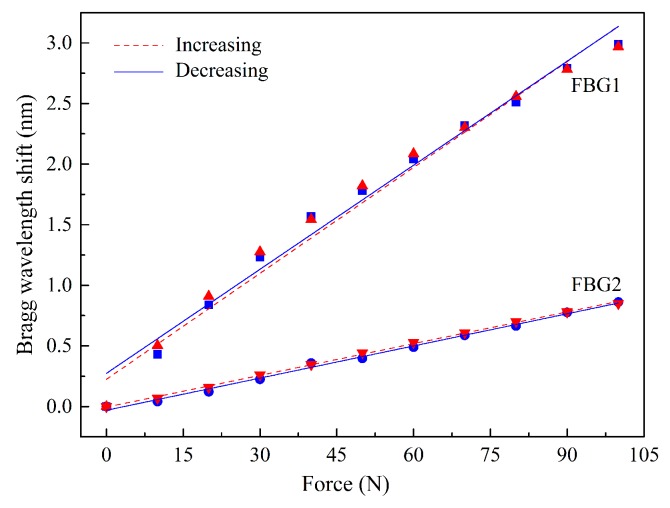
Bragg wavelength shifts as function of increasing and decreasing loadings.

The results obtained for the hysteresis tests are presented in Figure 5. The maximum values found were 0.07 nm for FBG1 and 0.05 nm for FBG2.

### 3.4. Implementation: Simultaneous F_V_ and F_S_ Loadings

After the calibration, and using the same experimental setup as depicted in Figure 2, the sensor was tested for simultaneous F_S_ and F_V_ loadings. The Bragg wavelength shifts, modulated in the optical fiber sensors under simultaneous shear and vertical loadings, can be related to the applied forces by a two-equation system [8]:

(2) $$\left[\begin{array}{c} F_{V}\\ F_{S} \end{array}\right]={\left[\begin{array}{cc} K_{1V} & K_{1S}\\ K_{2V} & K_{2S} \end{array}\right]}^{-1}\left[\begin{array}{c} \Delta \lambda_{FBG1}\\ \Delta \lambda_{FBG2} \end{array}\right]\;⬄\;\left[\begin{array}{c} F_{V}\\ F_{S} \end{array}\right]={\left[\begin{array}{cc} 14.15\times 10^{-3} & -26.02\times 10^{-3}\\ 7.35\times 10^{-3} & -24.29\times 10^{-3} \end{array}\right]}^{-1}\left[\begin{array}{c} \Delta \lambda_{FBG1}\\ \Delta \lambda_{FBG2} \end{array}\right]$$

where Δλ_FBG1_ and Δλ_FBG2_ are the Bragg wavelength shift of FBG1 and FBG2, respectively.

**Figure 6:**
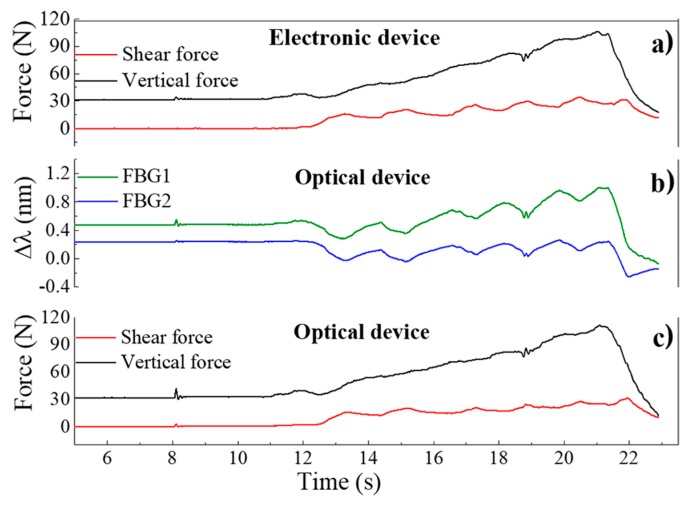
Response to the applied forces as a function of time for the: (**a**) electronic sensor; (**b**) FBGs cell, with the response as Bragg wavelength shift; (**c**) FBGs cell, the forces are calculated by applying Equation (2) to the registered wavelength shifts.

In Figure 6, the values acquired for the electronic and optical sensing units during this test are presented, as well as the data acquired after the application of Equation (2). The plot in Figure 6a corresponds to the values registered by the 3-axial electronic force sensor, while the data depicted in Figure 6b are the corresponding Bragg wavelength shift values acquired through the optical sensing cell.

**Figure 7:**
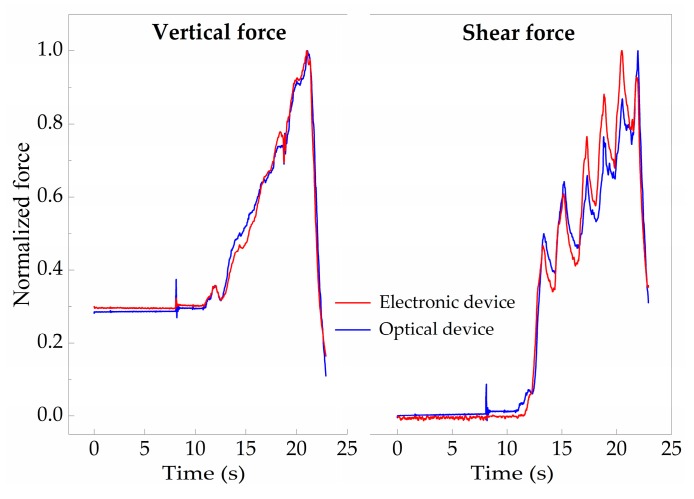
Comparison between the normalized values acquired with the three-axial sensor and the FBGs cell (RMSE_V_ = 0.025 and RMSE_S_ = 0.053).

After applying Equation (2) to the Δλ values obtained from the optical sensing unit, it is possible to obtain the correspondent force values, as presented in Figure 6c, which match the values acquired by the electronic sensors. Moreover, when comparing both sensors’ responses, the differences between the curves obtained by the optical and the electronic sensor have a normalized root mean square error value of RMSE_V_ = 0.025 for F_V_ and RMSE_S_ = 0.053 for F_S_, as presented in Figure 7, indicating the reliable performance of the optical sensor and its suitability to monitor F_V_ and F_S_, simultaneously.

The compactness, accuracy and reliability of the presented solution is demonstrated in shoe insoles for non-invasive gait pattern analysis (Section 4), however, its application in rehabilitation exoskeleton robots has great potential and will also be considered in our future work.

## 4. Gait Simultaneous Shear and Plantar Pressure Monitoring

In this section, we present the integration of the sensing architecture described before in an insole for the continuous and simultaneous monitoring of shear and plantar pressure during gait. This proposed solution is compact in size, non-invasive and could be used continuously during a daily routine, without jeopardizing the mobility of patients nor their autonomy while providing an early assessment of the gait pattern abnormalities of individuals at risk.

### 4.1. Insole Design and Implementation

Considering the foot plantar anatomy, the most at risk areas to develop neuropathic ulcers are the regions covering the bony prominences, where the load is heavily applied. Such areas are located under the metatarsal heads. Nonetheless, the shear stress measured in the great toe and heel are also reported as key points of analysis [6].

During gait, the value of vertical reaction forces and anterior-posterior (AP) forces (shear) are related to the body weight. As for vertical forces, a typical maximum (over the plantar area) is obtained with a force corresponding to 120% of the body weight at the early stance and toe-off moments [36,37]. For the anterior posterior forces, they are considerably smaller and can reach up to 25% of the body weight [36,37]. Locally wise, the area in which the vertical force will have a higher amplitude is the heel area, with a force that can reach up to 80% of the body weight [37].

**Figure 8:**
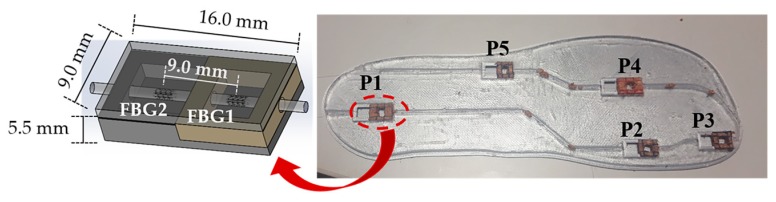
Photograph of the insole used for shear and plantar pressure monitor, incorporating five FBGs cells (as also schemed).

Bearing in mind such key areas and gait pattern features, we have designed and produced an insole incorporating a total of five sensing cells, similar to the one described in Section 3. A single optical fiber, containing a total of 10 FBGs, was incorporated in the insole, as depicted in Figure 8.

The FBGs cells were placed in the key points of analysis for the foot plantar pressure and shear stress monitor, namely, heel (P1), midfoot (P5), metatarsal (P2 and P4) and toe (P3). The Bragg wavelength and grating periodicity for each FBG are presented in Table 1.

### 4.2. Shear and Plantar Pressure Results

With the developed system, by monitoring the wavelength shift experienced by the FBGs in each cell, it is possible to simultaneously monitor the patient’s gait pattern, as well as the plantar pressure (corresponding to the vertical force mention in previous sections, for unit of area) and shear stress distribution. Nonetheless, prior to its dynamic application, it is necessary to calibrate each sensing cell to pressure and shear. In that way, the procedure described in Section 3.2 was performed individually for each sensing cell, from which the sensitivities values K_1V_, K_2V_ (to vertical forces) and K_1S_, K_2S_ (to shear) were obtained for each FBGs cell.

**Figure 9:**
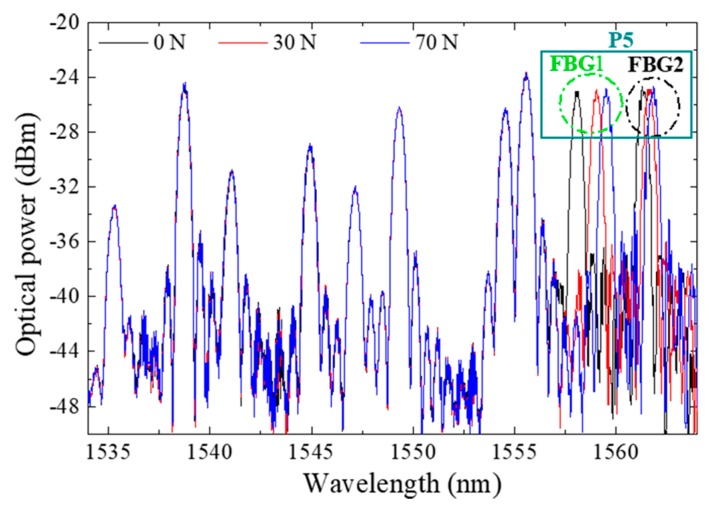
Optical spectra obtained during the pressure calibration of FBGs cell “P5”.

Figure 9 displays the optical spectra obtained for the developed insole during the vertical force calibration of the FBGs cell “P5”. Since the whole system is placed in one optical fiber with 10 multiplexed FBGs, the Bragg wavelength corresponding to each FBG is also visible. However, during the calibration process, only the FBGs corresponding to the sensing cell inserted in P5 responds to the local applied loads. Such a characteristic confirms the good isolation of the designed sensing cell to forces applied in its surroundings, and its suitability for a precise local analysis. Additionally, the presented design (size, number and location of the sensing cells) can be customized according to a doctor prescription and each patient/situation’s specific needs.

After calibration, the insole was placed in a shoe for a dynamic monitoring test. During the test, the interrogation system was continuously acquiring the Bragg wavelength shift registered in each point, while the subject (female with 45 kg) was walking.

**Figure 10:**
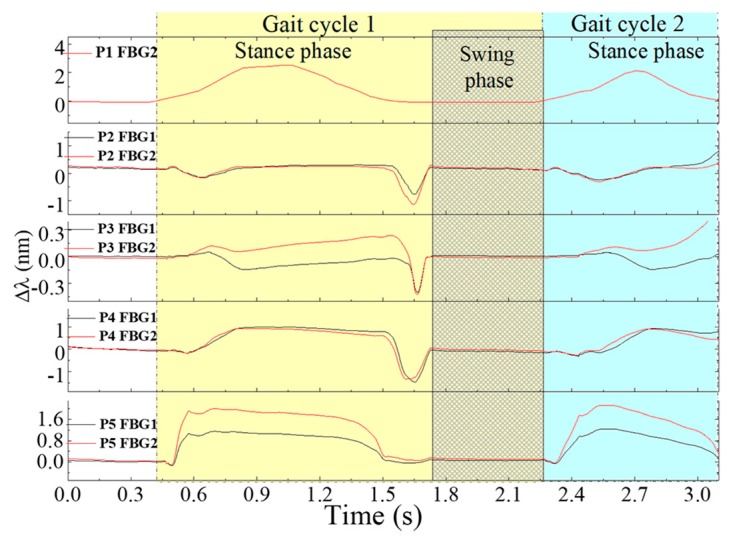
Bragg wavelength shifts in time, registered for each FBG in the 5 points of analysis.

Figure 10 represents the registered Bragg wavelength shift for each FBG, in the 5 points, during a 3 s gait. Due to a malfunction in the interrogation system, the Bragg wavelength acquired for FBG1 in P1 had extensive value gaps along time (Bragg wavelength returned as zero) and therefore its values were ignored.

For the global set of the remaining sensing cells, it is clear that each point is activated according to the pressure pattern expected during gait. The stance phase (foot in contact with the floor) and swing phase (foot without contact with the floor) are also clear in this representation [3].

Moreover, the instant in which the shear is a dominant force in the gait cycle is clearly visible (end of the stance phase), as well as the areas in which it is more predominant, namely, the metatarsal (P2 and P4) and the toe (P3) areas.

**Figure 11:**
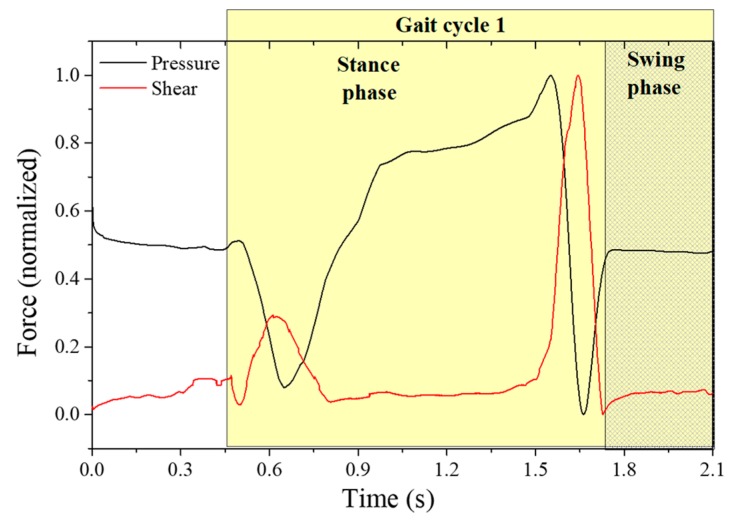
Plantar pressure and shear stress retrieved from the sensing cells in the insole during gait.

In order to retrieve the values of plantar vertical and shear forces from the raw data plotted in Figure 10, we apply Equation (2) to each point. The resultant curve, obtained for point 2, located at one of the metatarsal heads (critical point for shear analysis), is depicted in Figure 11.

As it can be seen, it is possible to differentiate the plantar vertical force and the AP shear stress curve, as reported previously [3,6]. From the obtained curves, it is also observed that the maximum shear stress occurs first in the beginning of the foot-flat phase and again, with higher intensity, at the rising of the heel and the toe-off phase, which corresponds to the backward acceleration force under the metatarsal areas [6].

It is worth noting here that the shear stress evaluated with the referred system is the AP longitudinal shear, and to evaluate the medial-lateral stress, a different sensing cell configuration should be designed. However, and as stated before (and shown in Figure 9), lateral forces do not affect the proposed sensing cells performances for AP shear stress and plantar pressure. Also, it should be noticed that the estimated dynamic range can reach up to at least ~350 N (considering the sensor at heel area, where a typical vertical force of 80% of the body weight is applied).

So, the designed system presented in this paper is a reliable solution for the simultaneous monitoring of plantar pressure and shear stress during gait. Although the developed insole is composed of resin, which is a semi-rigid material, due to its flexibility and the small thickness used for this application, it is possible to be integrated inside the orthopedic insoles, for more comfortable wear. Its application as e-Health tool can provide a clear advantage to patients prone to develop neuropathic ulcers, by early alerting them to correct their posture and walking pattern [4,38]. Also, the incorporation of such devices in rehabilitation exoskeletons will allow the mitigation of the existing gap regarding the monitoring of shear forces.

## 5. Suitable Non-Invasive e-Health Solution

**Figure 12:**
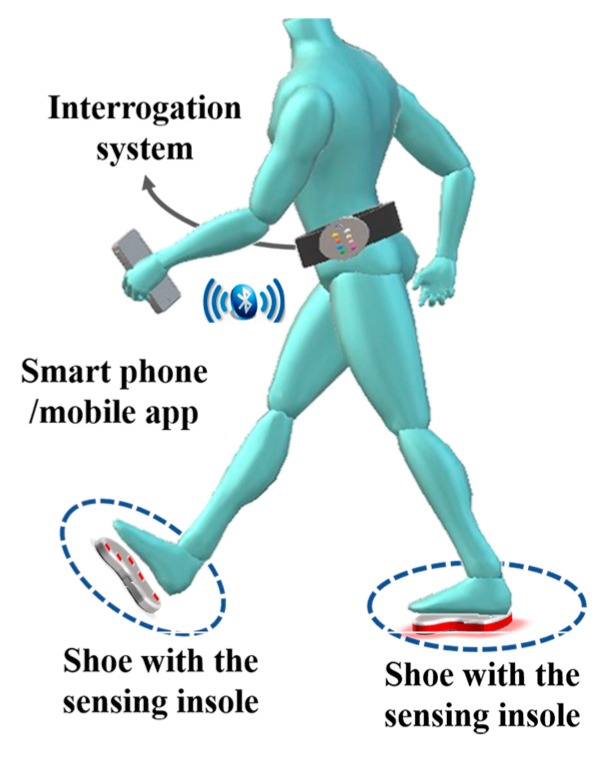
Non-invasive e-Health optical fiber sensing architecture for shear and plantar pressure gait analysis.

It has been emphasized throughout this paper the importance of the compact size of the sensing architecture, in addition to its resilience. These two properties render the architecture suitable for integration within a non-invasive e-Health system for the continuous monitoring of patients and elderly citizens. The proposed insole design, described in Section 4, would comprise one part of the whole mobile e-Health solution, used to continuously monitor patients for irregularities in their gait movement. The envisioned overall non-invasive monitoring solution is shown in Figure 12. The system comprises three components: the sensing element, the interrogator system, and the mobile app on a smartphone. The first part, which is the optical fiber sensing architecture, has been extensively explained throughout the paper. It is basically represented by the insole integrated with the optical FBGs cells, as explained in previous section.

The second part is the interrogator system, required to acquire the signal modulated in the sensing points. This can be translated into shear and vertical pressure, based on the proposed system of two equations, as explained in Section 3.

The last component is a mobile app installed on a smartphone. The mobile app has multiple roles. First, it is used as a data processing tool to analyze the acquired measurements from the sensing system. Second, the app uses the smartphone as a gateway for transmitting the measured data to the cloud, as shown in our previous work [3]. Additionally, the app can be used to display the results, when required by the patient. The user (patient or his/her doctor) is able to use the app to view the statistics from the measured data over a period of time. Although the system is continuously measuring the pressures, the main requirement is not the instantaneous display of measured values, but the average overall performance of monitored patients/elders during their actual daily routines. Hence the results are continuously transmitted to the cloud for a more elaborate analysis by medical personnel (i.e., the doctor, nurse or physical therapist), eliminating the requirement for high computational and energy capabilities at the mobile device [38]. It is worth mentioning here that the overall e-Health solution is still under development and results obtained from the system will be presented in future work.

## 6. Conclusions

In this work, a novel compact and efficient optical fiber based solution for the simultaneous sensing of vertical and shear forces is presented. The proposed architecture is accurate and resilient compared to existing solutions. The results obtained from the developed sensing cell show similar behavior to the three-axial electronic force sensor used for comparison, with a RMSE_V_ = 0.025 and RMSE_S_ = 0.053 between them. These values show that the developed device achieved the necessary accuracy while offering all the optical fiber sensor technology advantages, like immunity to electromagnetic interference and humid environments. Moreover, the proposed one-dimensional configuration is a reliable solution, which facilitates the production and incorporation of the sensing cell elements in other devices. Additionally, the presented sensing element, being able to infer and discriminate shear from vertical forces, has a great potential for incorporation into insoles for the measurement of plantar pressure (vertical force) and shear force. This measurement has high potential in different contexts/scenarios, including the prevention and study of pressure ulcers or in monitoring the performance of athletes during training; in electronic skin (e-skin) technologies; intelligent and rehabilitation robotic exoskeletons; human-machine interaction devices or even biomimetic prosthesis.

As for future work, it is our intention to optimize the cell to be able to retrieve both shear forces, anteroposterior and medial lateral, and validate its functionality within several individuals of both genders and of different age groups. Furthermore, its integration in an overall non-invasive e-Health architecture is being assembled, which will allow us to evaluate the forces during gait remotely and in a real time, enabling the monitoring of patients and elder citizens during their active lifestyle routines, without jeopardizing their mobility or freedom.

## Figures and Tables

**Table 1:** Bragg wavelength and grating periodicity for each FBG.

		Bragg Wavelength (nm)	Grating Period (nm)
**P1**	FBG1	1535.1	522.1
	FBG2	1547.1	526.2
**P2**	FBG1	1540.4	524.0
	FBG2	1543.8	525.1
**P3**	FBG1	1549.2	527.0
	FBG2	1555.7	529.1
**P4**	FBG1	1537.1	522.8
	FBG2	1552.8	528.2
**P5**	FBG1	1557.9	529.9
	FBG2	1561.4	531.1
